# Correction: Non-Linear Analysis Indicates Chaotic Dynamics and Reduced Resilience in Model-Based Daphnia Populations Exposed to Environmental Stress

**DOI:** 10.1371/journal.pone.0107090

**Published:** 2014-08-25

**Authors:** The PLOS ONE Staff

## Notice of Republication

This article was republished on August 7, 2014, to replace incorrectly changed character in the byline, citation, and references. The publisher apologizes for the errors. Please download this article again to view the correct version. The originally published, uncorrected article and the republished, corrected article are provided here for reference.

## Supporting Information

File S1 Originally published, uncorrected article.
(PDF)

File S2 Republished, corrected article.
(PDF)
