# Supplementary material for: Non-Linear Analysis Indicates Chaotic Dynamics and Reduced Resilience in Model-Based Daphnia Populations Exposed to Environmental Stress
Source: PLoS One. 2014 May 8;9(5):e96270. doi: 10.1371/journal.pone.0096270 (PMC4014494; doi:10.1371/journal.pone.0096270)
Supplement: MATLAB source code S1 — Non-linear time series analysis using the Tisean package. (DOC) [file pone.0096270.s006.doc]

**MATLAB source code for non-linear time series analysis**

% Load Tisean

tiseanPath = '/home/user/Tisean_3.0.0/bin/';

% Load data

x=load('/home/user/data.dat');

a=x';

% Plot time series

for i= 2 : 101

subplot(10,10,i-1);

plot(x(:,i),'k');

end

% Mean values per data set

mean_x=mean(x);

% Length of data vector

length_x=length(x);

% Histogram

for i = 2 : 101

subplot (10, 10, i-1)

hist(x(:,i),100,'k');

end

% Autocorrelation

for i = 2 : 101

system([tiseanPath, 'corr /home/user/data/data.dat -c',num2str(i),' -o /home/user/data/test.cor']);

corrdata=load('/home/user/data/test.cor',' ',2);

subplot (10, 10, i-1)

plot(corrdata(:,1),corrdata(:,2),'k')

end

% Recurrence plot

for i = 2 : 101

system([tiseanPath, 'recurr /home/user/data/data.dat -c',num2str(i),' -o /home/user/data/test.rec']);

rec=load('/home/user/data/test.rec');

subplot (10, 10, i-1)

scatter(rec(:,1),rec(:,2),'ok','filled');

end

% Time delay

for i = 2 : 101

system([tiseanPath, 'delay -c',num2str(i),' -d8 -o /home/user/data/delay1.data /home/user/data/data.dat']);

y1=load('/home/user/data/delay1.data');

subplot(10,10,i-1)

plot(y1(:,1),y1(:,2),'.-k')

end

% Lyapunov-Exponent k

for i = 2 : 101

system([tiseanPath, 'lyap_k -c',num2str(i),' -M6 -m3 -d8 -t100 -s500 -r.1 -o /home/user/data/lyap_k',num2str(i),'.data /home/user/data/data.dat']);

end
