# Supplementary material for: Correction: Non-Linear Analysis Indicates Chaotic Dynamics and Reduced Resilience in Model-Based Daphnia Populations Exposed to Environmental Stress
Source: PLoS One. 2014 Aug 25;9(8):e107090. doi: 10.1371/journal.pone.0107090 (PMC4143371; doi:10.1371/journal.pone.0107090)
Supplement: File S1 — Originally published, uncorrected article. (PDF) [file pone.0107090.s001.pdf]

PLOS ONE

Non-Linear Analysis Indicates Chaotic Dynamics and Reduced Resilience in Model-Based Daphnia Populations Exposed to Environmental Stress

Richard Ottermanns*, Kerstin Szonn, Thomas G. Preuβ, Martina Roβ-Nickoll

Institute for Environmental Research, RWTH Aachen University, Aachen, Germany

Abstract

In this study we present evidence that anthropogenic stressors can reduce the resilience of age-structured populations. Enhancement of disturbance in a model-based Daphnia population lead to a repression of chaotic population dynamics at the same time increasing the degree of synchrony between the population's age classes. Based on the theory of chaos-mediated survival an increased risk of extinction was revealed for this population exposed to high concentrations of a chemical stressor. The Lyapunov coefficient was supposed to be a useful indicator to detect disturbance thresholds leading to alterations in population dynamics. One possible explanation could be a discrete change in attractor orientation due to external disturbance. The statistical analysis of Lyapunov coefficient distribution is proposed as a methodology to test for significant non-linear effects of general disturbance on populations. Although many new questions arose, this study forms a theoretical basis for a dynamical definition of population recovery.

Citation: Ottermanns R, Szonn K, Preuβ TG, Roβ-Nickoll M (2014) Non-Linear Analysis Indicates Chaotic Dynamics and Reduced Resilience in Model-Based Daphnia Populations Exposed to Environmental Stress. PLoS ONE 9(5): e96270. doi:10.1371/journal.pone.0096270

Editor: Jordi Garcia-Ojalvo, Universitat Pompeu Fabra, Spain

Received December 13, 2013; **Accepted** April 4, 2014; **Published** May 8, 2014

Copyright: © 2014 Ottermanns et al. This is an open-access article distributed under the terms of the Creative Commons Attribution License, which permits unrestricted use, distribution, and reproduction in any medium, provided the original author and source are credited.

Funding: The authors have no support or funding to report.

Competing Interests: The authors have declared that no competing interests exist.

* E-mail: ottermanns@bio5.rwth-aachen.de

Introduction

Recovery, resilience and disturbance

Ecosystems are increasingly affected by human activity. Structural as well as chemical impacts threaten ecological populations, communities, the connected ecosystem functions, processes and services [1]. The questions how the constituents of ecosystems react to those impairments and whether they can recover are matter of many ecological research studies (e.g., [2], [3], [4], [5], [6], [7], [8]).

The term recovery is often stressed in ecology and monitoring. In its broadest sense recovery is defined as the recurrence of a system to a preceding status after disturbance, often quantified as the time to achieve this pre-disturbance status. Thereby the concept of recovery is used to quantify how severely a system is affected by a disturbance but also to assess whether a system is enduringly negatively affected.

Recovery constitutes a part of the much debated concept of ecological stability which is connected to various other ecological aspects as resilience [9], [10]. Like for recovery different definitions of the term resilience exist (e.g., [11], [12]). Engineering resilience is defined as the time that a system takes to return to equilibrium following perturbation [13], whereas ecological resilience is defined as the amount of perturbation a system can withstand before it moves into a different basin of attraction or stability domain [14]. Often resilience is simply defined as the capacity of the system to return to a given state after a disturbance [15]. In this last case recovery and resilience are used synonymously (resilience = rate of recovery).

As resilience reflects the ability of a community or a population to withstand and recover from disturbance it is assumed a fundamental property of ecological systems and thus has been proposed as an indicator of ecological health and integrity [16]. Within this context disturbance is broadly defined as any relatively discrete event that disrupts ecosystem, community, or population structure and changes resources, substrate availability or the physical environment [17].

It is generally accepted that most species communities are constantly exposed to natural disturbance resulting in changes of community structure and life history characteristics of the constituting species. From the idea that many of the environmental parameters that determine resilience of populations and communities to natural disturbance may also influence the response to chemical stressors [16] it can be concluded that exposure to contaminants or other anthropogenic impacts alters the ability to withstand and recover from natural disturbance via reduced resilience.

Two different types of disturbance can be distinguished. On the one hand pulse disturbance [18] results in prompt change in species abundance within a community (e.g. forest fire or chemical spill that reduces densities of certain species). Effects of pulse disturbances are generally assumed to recede after some time so that the system can return to its initial equilibrium state. On the other hand press disturbance causes permanent change in species abundance and often leads to the loss of some taxa and establishment of an alternative community (e.g. increased temperature associated with global climate change or continuous input of toxic material into a system). Until the elimination of the

stressor a community under continuous press disturbance is generally not accepted to return to its original condition [16].

Dynamics of age-structured populations

The fact that age structure is an important feature of natural populations has been deduced in many studies based on population models [19], [20], [21]. Many animal species, like crustaceans, have life-histories composed of a sequence of stages within which their individuals' characteristics are broadly similar to those of other individuals in the same stage and markedly different from those of individuals in other stages [22]. Within these complex life-cycles the vital rates (rates of survival, development, and reproduction) almost always depend on individuals' age, size, or development stage. It has become widely recognized that this diversity can have a significant influence upon dynamics at the population level and that in order to understand the dynamics of most populations it is necessary to take this diversity adequately into account [23], [24].

Such variation can be described with the help of time discrete nonlinear age-structured population models (for examples see [25], [26], [27], [28], [29], [30]) which have been widely studied in the biomathematical literature [31] (e.g., [32], [33], [34], [35], [36], [37], [38]). A large number of theoretical analyses and numerous applications to ecological systems have proven the relevance of age-structured population models (e.g. [22], [28], [31], [39], [40], [41], [42], [43], [44], [45], [46]).

Even though these models are of simple structure it was shown that they display all kinds of dynamical behaviour, like equilibria, periodic cycles, and unpredictable chaotic dynamics [40], [47], [48], [49], [50], [51], [52], [53], [54], [55]). The occurrence of this complex dynamic behaviour is related to the fact that minor changes in parameter or initial values can strikingly change the dynamic behaviour of systems [31]. Model dynamics were found to be very sensitive to changes in life-history parameters as well as environmental perturbations [28]. In more general terms of system dynamics it was shown that trends and fluctuations in populations are determined by complex interactions between extrinsic forcing and intrinsic dynamics [46].

Daphnia magna as model organism

Daphnia magna is a Crustacean in the order of Cladocera. In terms of assessing the effects of human contaminants *D. magna* exhibits an ideal test organism for this purpose as it constitutes a central component in aquatic food web structure [56], [57], [58] and is known to be sensitive to a multitude of xenobiotics (organophosphates, heavy metals, organochlorines, pyrethroids etc.) [59], [60], [61]. Due to its ease of culture and its clonal method of reproduction [56], [62] it is commonly used as a model organism in ecological risk assessment of pesticides, biocides, and industrial chemicals [29].

Within a *Daphnia* population three age classes (roughly equal to size classes) representing the different life stages can be distinguished, defined by means of age and fertility: neonates (less than 24 h old), juveniles (at least 24 hours old but non-mature), and adults (mature). These age classes differ in terms of their toxicological sensitivity in mortality, reproduction and developmental time. Effects within the population structure due to xenobiotics emerge from these age class specific sensitivities, e.g. reproduction rate.

Such a daphnid population exhibits a simple example of an ecological system with multi-stage interactions of age-classes a_n, $n = \{1, 2, 3\}$. Due to its reduced dimensionality and the differential interactions within it, the population comes close to the classical example of a 3-dimensional dynamical system like the Lorenz attractor. This suggests to analyse the effects of disturbance (effect = altered intrinsic rate of natural increase) of this system in terms of non-linear dynamics.

DCA as a model substance

3,4-Dichloroaniline (DCA, CAS-Nr.: 95-76-1, EEC-Nr.: 202-448-4) is an intermediate used for the synthesis of plant protection products (e.g., linuron, diuron, propanil) and as an azo dye for polyester fabrics. Release into the environment mainly occurs during use of plant protection agents after biotransformation or simply as an impurity of these substances [63]. DCA is known to affect survival and reproduction in *D. magna* already in low concentrations, resulting in reduced offspring number and increased number of aborted eggs in chronic toxicity tests [64], [65], [66]. In higher concentrations DCA has a lethal effect, causing increased mortality rates in acute toxicity tests [67]. Thus a strong effect of DCA on *Daphnia* population dynamics is obvious [68] and it exhibits a useful model substance to investigate effects on population level.

Non-linear time series analysis

The autocorrelation function allows for determining the non-whiteness of data and detecting periodic components in data. Because the autocorrelation function and the power spectrum are related by the Fourier transform, they are mathematically equivalent, and contain the same information [69].

Based on Takens' theorem [70] time-delay embedding allows for the reconstruction of the system's phase space and its attractors [71], [72], [73]. Scalar observations $x(n)$ and their time delays $x(n + kT)$; $k = 1,2,\ldots,m-1$ are used to make m-dimensional vectors $y(n) = [x(n),x(n+T),\ldots,x(n+(m-1)T)$ whose components provide a coordinate system in which one can identify the attractor structure associated with the observations ($T =$ time delay)[74].

Recurrence plots are a graphical tool for measuring the time constancy of dynamical systems [75]. They represent the recurrence of the phase space trajectory to a certain state, which is a fundamental property of deterministic dynamical systems [76], [77]. The analysis of recurrence plots allows for the detection of periodicity and regression [75] as well as typical transitions, e.g., bifurcation points occurring in complex systems [78].

A Poincaré section (also called a surface of section, Poincaré map or first recurrence map) [79] creates a projection of intersection points of an attractor and a hyper-plane in phase space [77]. It differs from a recurrence plot in that space, not time, determines the plot points. The hyper-plane is adjusted to cut the attractor transversal, thereby depicting the orientation of the n-dimensional attractor as a cloud of points in (n-1)-dimensional space.

Lyapunov exponents (λ) are a measure for exponential divergence of initially nearby trajectories in embedded phase space and are thus a hint for chaotic dynamics within a dynamical system [80]. If a dynamical system exhibits at least one Lyapunov exponent larger than zero it is assumed to show sensitivity to initial conditions, a prerequisite for chaotic dynamics [81], [82]. Calculating maximal global Lyapunov coefficients instead of local coefficients has the advantage of averaging out local divergence rates [83].

Aims

In this paper the authors present an analysis of the dynamics of a model-based age-class structured population of *Daphnia magna* exposed to different concentrations of a chemical stressor (3,4-Dichloroaniline, DCA). To support the understanding of the effects of chemical disturbance the study focused on the non-linear

characteristics of these dynamics and the question whether there is evidence that disturbance may lead to non-reversible alterations of population dynamics. This was achieved by quantification of periodic and chaotic parts of population dynamics, using methods from non-linear analysis and concepts from dynamical systems theory.

The hypothesis to be tested in this study was that anthropogenic stressors can alter the resilience of an age-structured population and thus have a negative influence on the ability to respond to further natural disturbance, thereby enhancing the risk of population extinction.

The Lyapunov was tested as an indicator to detect disturbance thresholds leading to a discrete change in the investigated population dynamics. Interpreting the results in terms of resilience, the study was intended to contribute to a dynamical definition of population recovery.

Materials and Methods

Model-based study

In principle there are three ways to reconstruct dynamics from non-linear systems: (1) analysis of system equations, (2) analysis of empirical data and (3) analysis of model-based data. In stress ecology simulation models are often used to predict recovery time [5], [29], [30], [84], [85], [86], [87], [88].

Analysis of system equations is more a theoretical task and not applicable in this context. Analysis of empirical data is often not optimal in the case at hand as the time series available is too short to analyze non-linear dynamics sufficiently and detect chaotic dynamics. Additionally empirical data are noisy and to find deterministic chaos the deterministic skeleton must be extracted from the data (which requires long data series as well). Thus, in this study we used a validated *Daphnia* population model (details see below) to simulate the empirical data. By this means we were able to (1) extend the observational time frame, as the model is validated to predict *Daphnia* population dynamics, (2) extract the deterministic skeleton, eliminating the confounding noise (we do this by setting chance constant) and (3) take into account the diversity within the complex life-cycle to understand the dynamics of *Daphnia magna* [23], [24].

Simulated data sets

The time discrete data used in this study were generated by use of an individual based population model for *Daphnia magna* (IDamP, [29]). The model predicts the population dynamics based on individual life cycles which include feeding rate, growth, development, reproduction and survival processes for which food conditions and population density (crowding effects) are the main drivers. The life cycle process is represented as descriptive regression models, based on a large dataset from *D. magna* life cycle tests.

IDamP reliably predicts population growth and reproduction for *D. magna* under laboratory conditions using the algae *Desmodesmus subspicatus* as a food source. It was recently shown to be able to extrapolate the individual effects of DCA to the population level as well as for other toxicants [30], [89], [90]. Besides mortality the only sub-lethal effects for DCA considered in the model is the inhibition of reproduction. The interactions between individuals are due to crowding and competition for food. To reduce the model stochasticity all individual properties, except a maximum lifetime assigned randomly to the initial individuals and the daphnids born during the simulation, were switched off. This maximum lifetime value was not changed anymore during the simulation. Raw data from the simulations can be obtained from the authors on individual request.

Five concentration levels for DCA were used in the simulations (0 μg/l, 2 μg/l, 5 μg/l, 10 μg/l, 20 μg/l, 40 μg/l), held constant over the simulation time frame of 365 days thus representing a press disturbance. Based on experimental laboratory data concentration-response relationships for DCA on the individual level had been calibrated in an integrated toxicity module in the IDamP model and validated on population level before [30].

As the DCA concentrations are far below the acute mortality concentrations for daphnids (48 h LC_{50}: 0.23 mg/l [63], [67]), it can be assumed that the effect will mainly occur on the adult daphnid reproduction rate.

A Monte-Carlo set of 100 simulation runs was conducted using the same parameter set as published in [30]. Differences in the 100 simulation runs resulted from differences in initial conditions. Before starting the simulations 5 neonate and 3 adult daphnids were introduced into the system. These 8 individuals were assigned random but fixed values for maximum lifetime.

As the chaotic parts of the population dynamics would be lost in non-linear analysis when based on mean values from these simulation runs, non-linear dynamical characteristics were calculated for each simulation run separately. Statistics were subsequently calculated from the resulting distributions of non-linear characteristics.

The overall population predicted by the model was additionally divided into three age classes (size classes): neonates (<1.4 mm), juveniles (≥ 1.4 mm and <2.6 mm) and adults (≥ 2.6 mm). In this way the data structure allowed for statistical analysis of population age class dynamics.

Modelling non-linear dynamics

Data analysis was based on the time series data described above. To exclude the effects from transient dynamics, the first 50 data points were omitted from all analyses. First, 3-dimensional phase space was plotted from the raw data of neonate, juvenile and adult daphnids. Secondly, exploratory analysis was performed on the time series data by use of linear autocorrelation functions. Finally, after embedding the time series, non-linear analysis was applied by means of recurrence plots, Poincaré sections and global maximum Lyapunov exponents. Data visualisation and analysis was performed by use of the software package Tisean [83], [91] in MATLAB (Ver. 8.2.0.701, release R2013b) on a Linux-based system (Debian testing Jessie). The MATLAB source code S1 is accessible from the supporting information.

In a 3-dimensional plot neonate, juvenile, and adult *Daphnia* abundances were plotted for each DCA-concentration. Trajectories were assessed in terms of compactness and orientation in phase space. Autocorrelation analysis was performed with function corr from the Tisean package to assess the amount of linear structures within the data. To find the best unfolding of the time series in the reconstructed phase space a series of time-delay embeddings was performed by means of the function delay from the Tisean package and the optimal parameters were chosen. Based on this assessment, finally all subsequent analyses were conducted with embedding dimension $m = 2$ and delay $d = 1$ (except the Poincaré section, which was performed with $m = 3$). Recurrence analysis [78] was performed to quantify the number and duration of recurrences of the system in its state space. We used the function recurr from the Tisean package, using $m = 2$ as embedding dimension, $d = 1$ as delay and $= 1$ as neighbourhood. Poincaré sections were created with the function poincare from the Tisean package applying an embedding dimension $m = 3$ and delay $d = 1$ to find a geometric depiction of the trajectories in a

lower-dimensional space. Quantification of attractor characteristics was done by calculation of global Lyapunov exponents. Maximum global Lyapunov coefficients for all 100 simulation runs per age class and concentration level were calculated with the function lyap k from the Tisean package [80], [81] using an embedding dimension m = 2 and delay d = 1, revealing a distribution of Lyapunov coefficients. Values larger than 1 were assumed to be a strong evidence for chaotic dynamics within the time series.

Statistical analysis

From the distributions of 100 Lyapunov coefficients mean values and standard deviations per age class and treatment level were calculated and tested for significant differences by means of a Wilcoxon signed-rank test (function wilcoxon.test in R, two.sided, conf.level = 0.95, [92]).

Results

All results are described for the overall population as well as the age classes (neonates, juveniles and adults). Figures are only shown for the overall population. The according figures for the age classes can be found in the supporting information.

The mean *Daphnia* abundance from all 100 simulation runs (including the 95% confidence interval) for the overall population in the control treatment is exemplarily shown as time series data in Fig. 1 (abundances for neonates, juveniles and adults can be found in the supporting information, Fig. S1). It was assumed that the population and its age classes existed under equilibrium conditions under control conditions from t = 50 on.

Population dynamics were quite comparable for control and DCA concentrations lower than 40 µg/l (Fig. 2). For the highest DCA concentration level the dynamics were obviously different.

In general the autocorrelation functions (Fig. 3) showed that with increasing DCA concentration no significant change in structure occurred. Comparable periodic dynamics of fluctuating correlation coefficients were observed up to 20 µg/l. This effect was especially strong for juveniles and adults, while in the neonate age class the effect was clearly less pronounced (see Fig. S2 in supporting information). In contrast the autocorrelation function for 40 µg/l showed a very strong correlation structure with clearly increased amplitude and decreased frequency.

From time-delay embedding (Fig. 4) it was concluded, that the trajectories for the overall population settled in the middle of the phase space. Especially for neonates and juveniles (see Fig. S3 in supporting information) the trajectories settled within a small part of the phase space nearer to the origin for the highest DCA concentration (40 µg/l).

Increased pattern formation was observed with increasing DCA concentrations in the recurrence plots (Fig. 5), especially for 40 µg/l. This was also observed for the age classes (see Fig. S4 in supporting information). For neonates a very strong lineal pattern

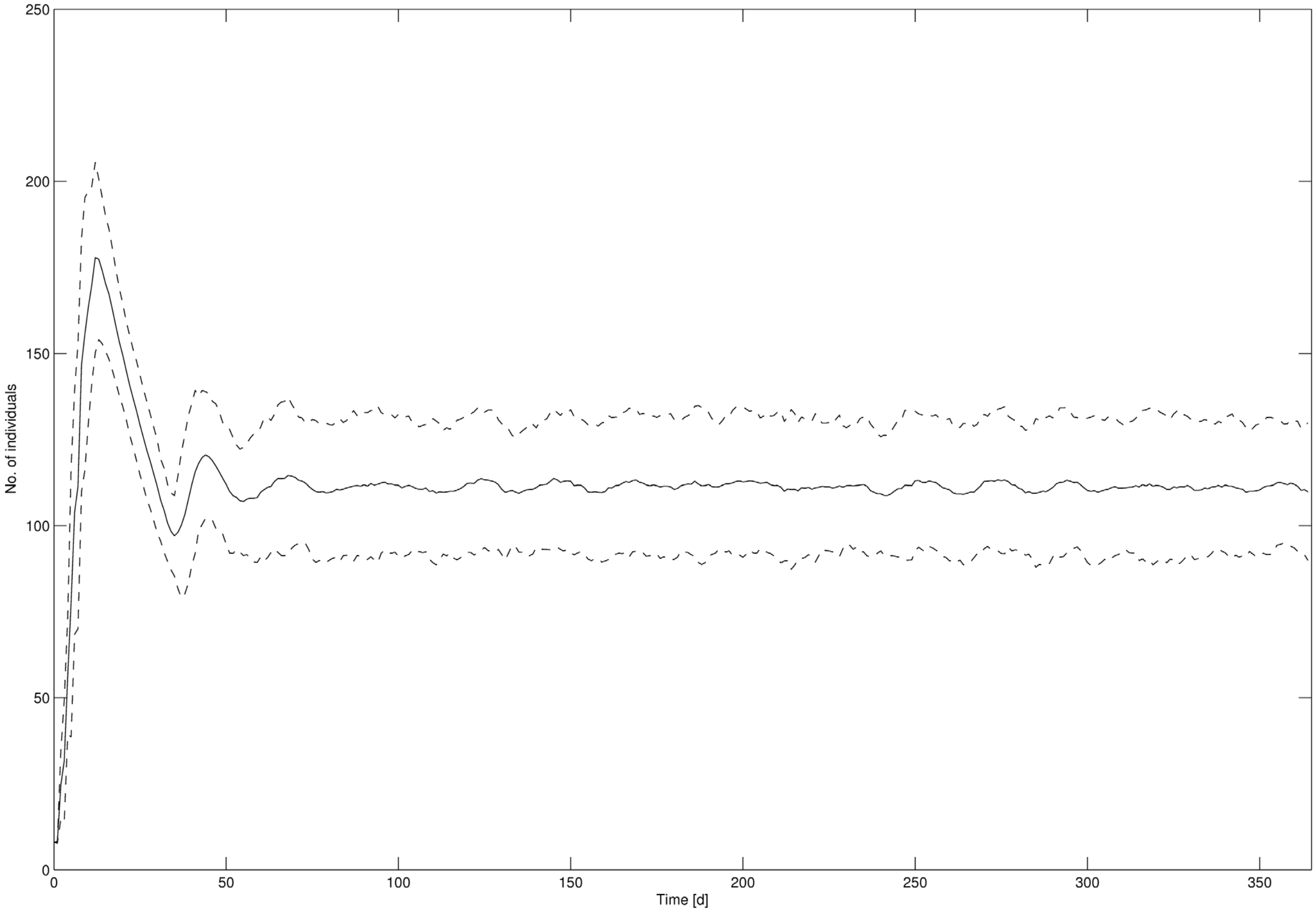

Figure 1. Daphnia abundance from all 100 simulation runs for the overall population in control. Solid line: mean abundance, dashed line: 95% confidence interval.
doi:10.1371/journal.pone.0096270.g001

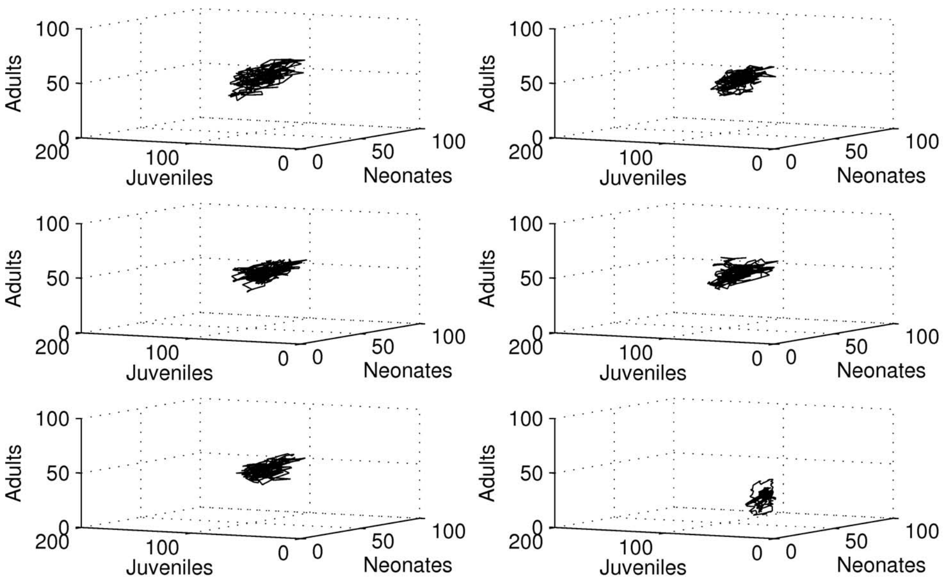

Figure 2. 3-dimensional phase spaces from raw data of simulation run 1 for neonates, juveniles and adults. From top left to bottom right, row-wise: control, 2 µg/l, 5 µg/l, 10 µg/l, 20 µg/l, 40 µg/l.
doi:10.1371/journal.pone.0096270.g002

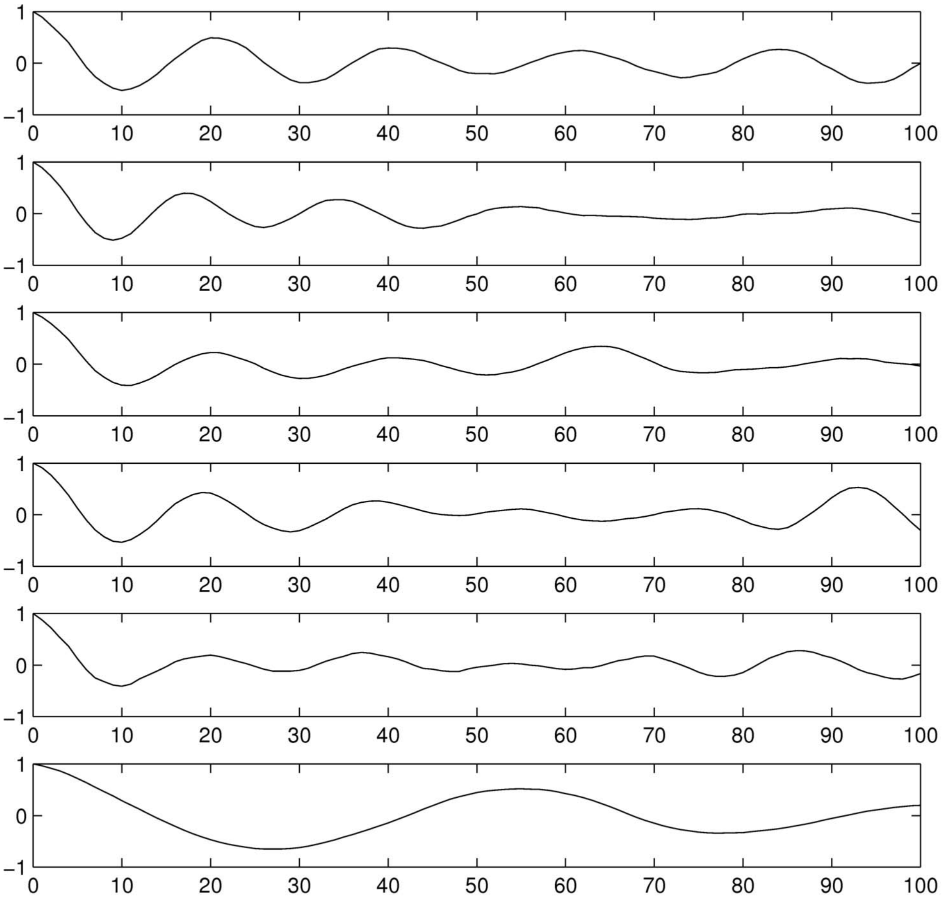

Figure 3. Autocorrelation functions of simulation run 1 for the overall population. From top to bottom: control, 2 µg/l, 5 µg/l, 10 µg/l, 20 µg/l, 40 µg/l, resp.
doi:10.1371/journal.pone.0096270.g003

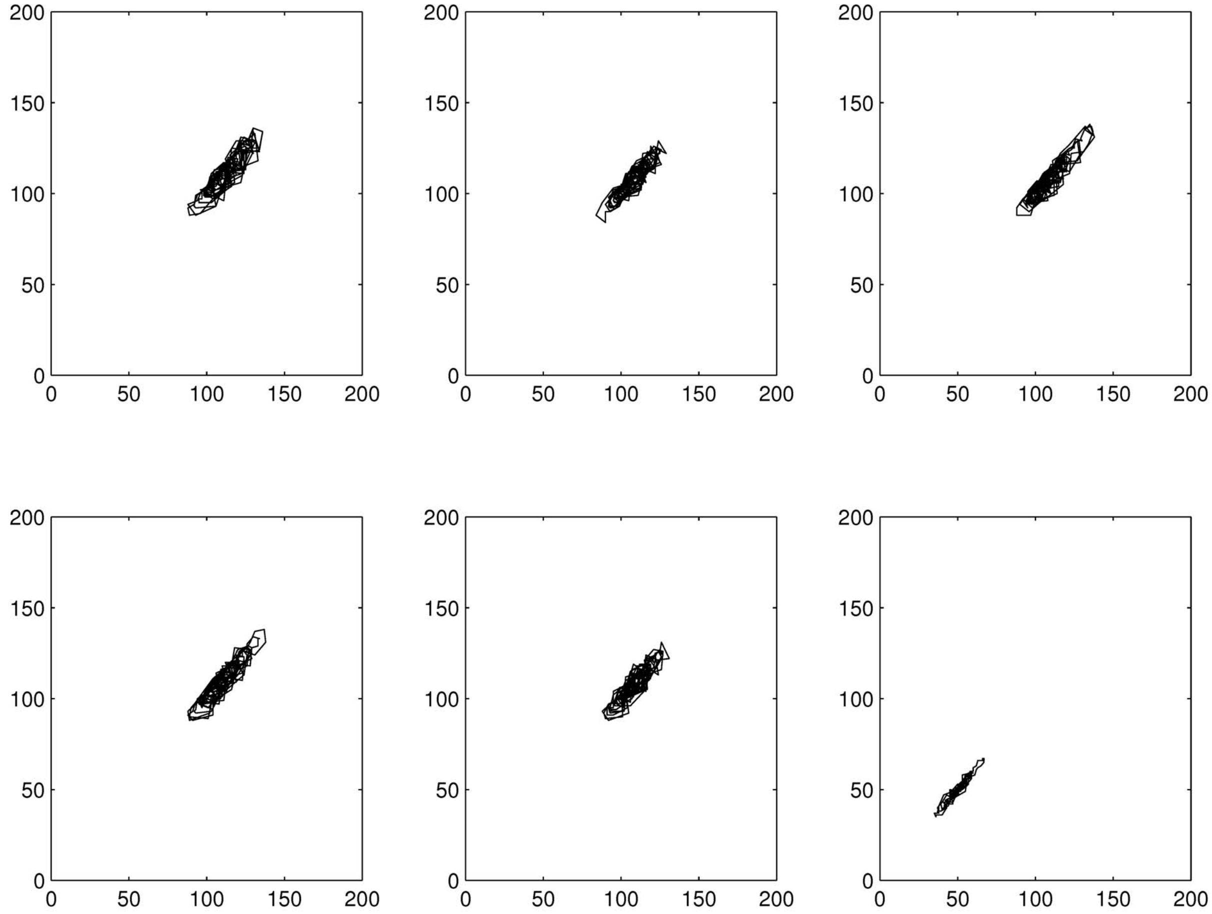

Figure 4. Embedded time series of simulation run 1 for the overall population. From top left to bottom right, row-wise: control, 2 µg/l, 5 µg/l, 10 µg/l, 20 µg/l, 40 µg/l, resp. (d = 1, m = 2).
doi:10.1371/journal.pone.0096270.g004

occurred in the highest concentration level, while for juveniles and adults this pattern formation was less pronounced. Especially for juveniles additional circular structures were observed in the highest concentration level.

Poincaré sections did not show large differences in dynamics for treatments from control up to 20 µg/l (Fig. 6). For 40 µg/l the attractor orientation in phase space abruptly changed in location (shift towards origin) and cross sectional area decreased. This effect was especially pronounced for juveniles and adults (see Fig. S5 in supporting information). For neonates the attractor was always located near the phase space origin.

Table 1 shows mean values and standard deviations for maximum Lyapunov coefficients calculated from the Lyapunov distribution for the overall population in the treatment levels. All maximum Lyapunov coefficients for all treatment levels were larger than zero (Fig. 7). Lyapunov coefficients of DCA treatments up to 20 µg/l were only slightly smaller than for control, while for 40 µg/l a large decrease was observed.

Table 2 shows the results from the Wilcoxon signed-rank tests on statistical differences between mean maximum Lyapunov coefficients for all treatment levels. The mean maximum Lyapunov coefficients for 40 µg/l were significantly different from all other treatment levels. Also, all mean maximum coefficients for the treatment levels were significantly different from the control.

Discussion

From the time series data in Fig. 1 it was concluded that under control conditions (no disturbance) the population and its age classes existed under equilibrium conditions. The existence of equilibrium is prerequisite in the attempt to define recovery because only if population structure is relatively stable, the population can return to defined pre-disturbance conditions if given sufficient time following the disturbance [16].

Generally the system seemed to converge to a quasi-stable state in the long run (see Fig. 2). For concentrations lower than 40 µg/l the system seemed to settle in a relatively small phase space region. This region occurred well bounded (a prerequisite for chaos), but the trajectory seemed to follow a complex attractor, exhibiting considerable population fluctuations. For the highest DCA concentration level fluctuations were much smaller compared to lower DCA concentrations. The trajectory settled within a much smaller phase space region, nearly approaching the zero abundance level, especially for the adult age class. These dynamics clearly exhibited an increased extinction risk for the overall population if this effect would have lasted too long (although this does not pose a problem to the age-class itself in the first place, as the classes are refilled by aging individuals).

For the overall population (as well as for all age classes) in the control and all treatment levels a sinusoid like oscillation was observed (see Fig. 3). Whether unpredictability results from a random process or is based on non-linear effects (deterministic

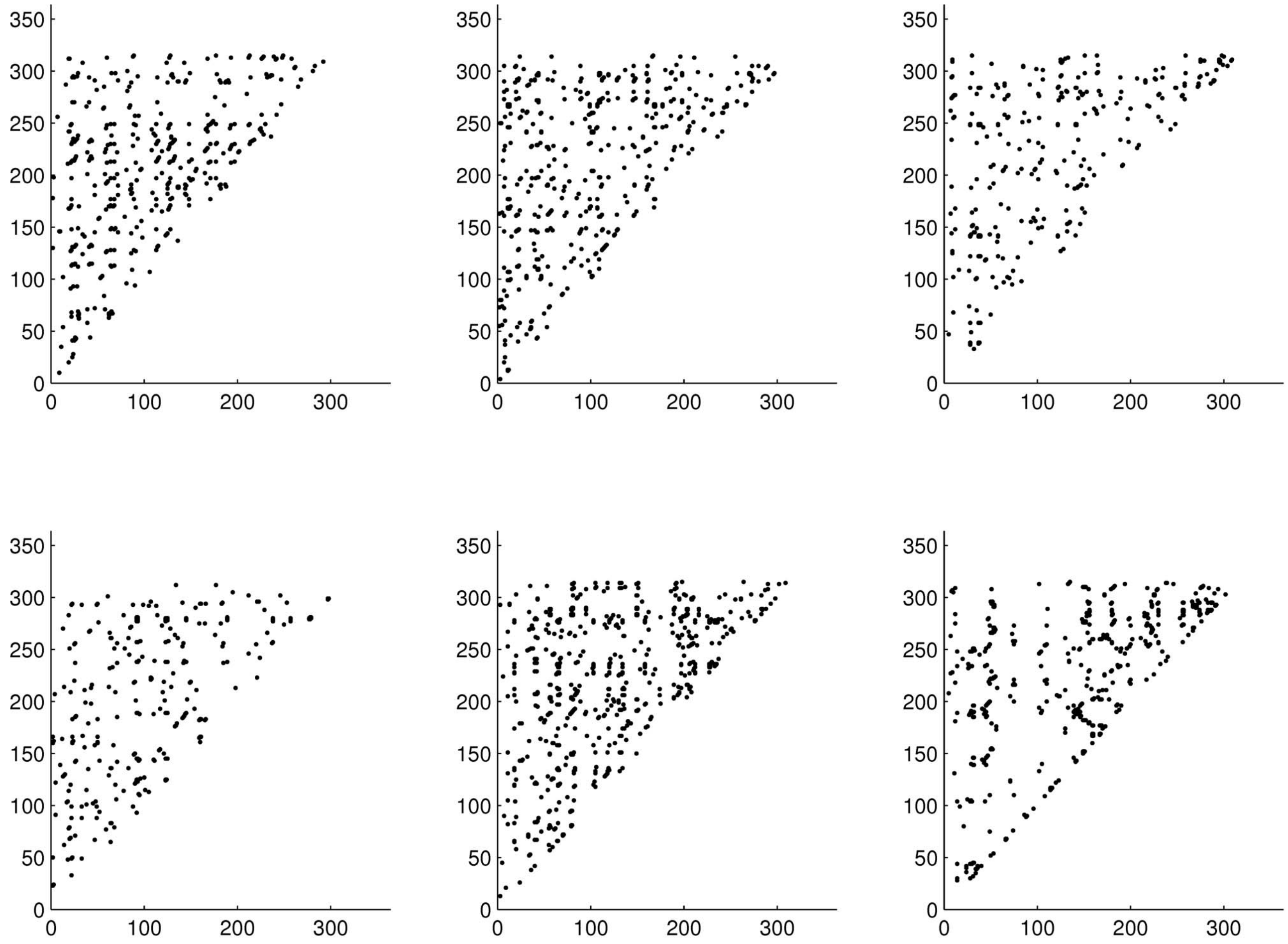

Figure 5. Recurrence plot of simulation run 1 for the overall population. From top left to bottom right, row-wise: control, 2 µg/l, 5 µg/l, 10 µg/l, 20 µg/l, 40 µg/l, resp. (d = 1, m = 1,2, ε = 1).
doi:10.1371/journal.pone.0096270.g005

chaos) can not be assessed by linear autocorrelation analysis as it is unable to distinguish between these two sources of fluctuation. However, since individual properties, except random individual maximum lifetime, were switched of to reduce the stochasticity of the model it is fare assumption that the unpredictability resulted exclusively from the non-linear effects.

For the highest concentration level the amplitude of the periodic oscillations clearly increased while the frequency decreased. This argues for an increased formation of long-range correlations within the data [93] for high concentration levels, enhancing the long-term predictability. It was thus concluded that the linear predictability of system dynamics increased with increasing disturbance.

As already observed for the raw data phase space, the system was forced towards an asymptotically stable equilibrium point for all DCA concentrations in embedded phase space (see Fig. 4). The fluctuations within the trajectory's settlement area decreased for the overall population and especially for the juvenile daphnids for the highest concentration level, reflecting the population's decreasing ability to respond to changes in environmental conditions (decreasing resilience).

Additionally, as the equilibrium point moved towards the phase space origin with increasing DCA concentration, the risk of extinction also increased.

The generally increasing pattern formation in the recurrence plots revealed, that the system dynamics increasingly tended to return to formerly engaged states when the disturbance increased (see Fig. 5).

While the more uniform spreading of points in the lower concentration levels suggested a high portion of chaotic dynamics (especially for the neonate daphnids), the lineal pattern found for in the highest concentration level suggested a high amount of periodic dynamics within the time series [94]. In contrast, the circular structures observed for juveniles in the highest concentration level suggested the occurrence of quasi-periodic dynamics, like superimposed harmonic oscillations. These structures can be suspected to originate from the realistic and model-built in time delay between juvenile response, their maturation and the according effects on reproduction.

For the juvenile daphnids a mixture-like pattern was observed in the highest concentration level. It seems like the system settled at a transition between quasi-periodic and periodic dynamics. Such a transition has already been observed in a population model for *Tribolium* [95], in which the varied parameter of disturbance was the rate of larvae developing to adults, resembling the effect on age class dependent mortality of juveniles within the *Daphnia* model. As already deduced from the autocorrelation function, from our findings it was concluded, that the predictability of the system dynamics increased with increasing disturbance.

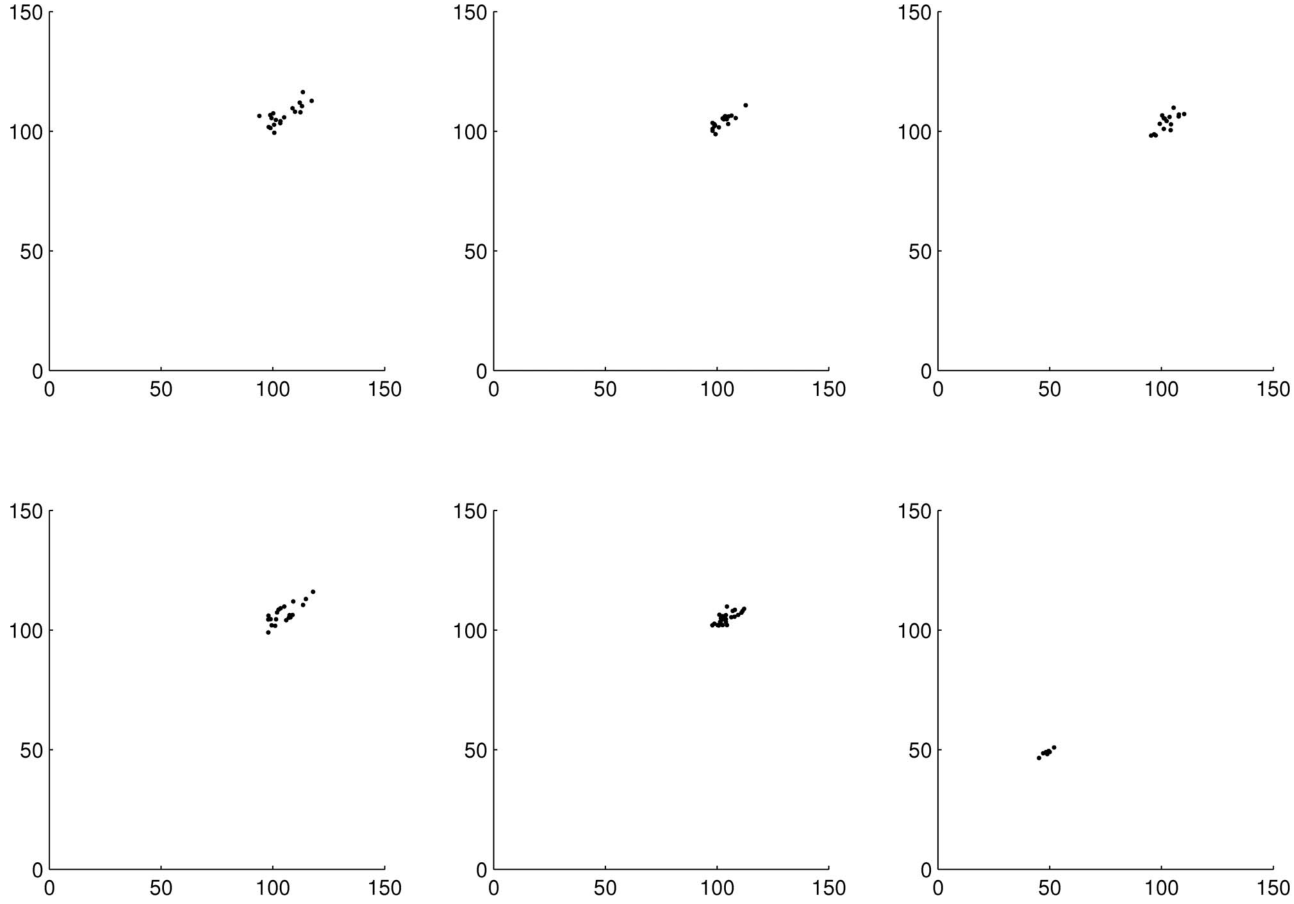

Figure 6. Poincaré sections of simulation run 1 for the overall population. From top left to bottom right, row-wise: control, 2 µg/l, 5 µg/l, 10 µg/l, 20 µg/l, 40 µg/l, resp. (d = 1, m = 3, C = 1).
doi:10.1371/journal.pone.0096270.g006

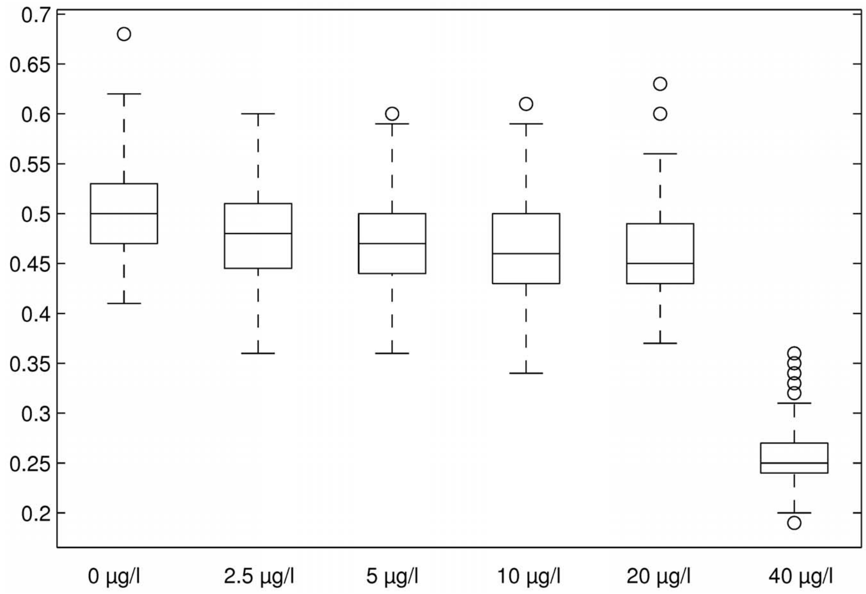

Figure 7. Boxplots for maximum Lyapunov coefficients for DCA treatments for the overall population. Boxes show mean value (horizontal line), 25 and 75% quantile (box edges), 5 and 95% quantile (whiskers) and outliers>2 sd from mean (circles).
doi:10.1371/journal.pone.0096270.g007

Table 1. Mean values and standard deviations for maximum Lyapunov coefficients per treatment level for the overall population with $n = 100$.

	Control	2.5 μg/l	5 μg/l	10 μg/l	20 μg/l	40 μg/l
mean	0.503	0.477	0.470	0.467	0.459	0.261
stdev	0.0471	0.0492	0.0485	0.0528	0.0507	0.0336

doi:10.1371/journal.pone.0096270.t001

The Poincaré sections illustrated clearly, that the attractor determining the system dynamics was more or less stable from the control up to 20 μg/l DCA in the overall population as well as all age classes (see Figs. 6 and S5). The abruptly changing location of the attractor (esp. for juveniles) suggested that the attractor became unstable between 20 μg/l and 40 μg/l.

As all Lyapunov coefficients (5 treatment levels ×3 age classes ×100 simulation runs = 1500 values) were larger than zero, and together with the fact that the dynamics were clearly bounded, there was strong evidence for chaotic dynamics within this model-based population of *Daphnia magna*. Consulting the Lyapunov coefficient as a measure of rate of chaotic dynamics within the population development, this rate decreased for DCA treatments compared to control (see Fig. 7). From the biological point of view this seems reasonable as positive feedback (influence of exponential growth) is likely to be smaller in the presence of a disturbance affecting reproduction.

The decrease of the coefficients indicated that higher amounts of disturbance decreased the potential for exponential increase of trajectory distances. This was already obvious from the former analyses, but the statistics on the coefficients quantified and validated this. It suggested some sort of concentration-response relationship, thereby approving the Lyapunov coefficient as an indicator to detect non-linear effects of population dynamic disturbance due to environmental stress. Additionally, the significant decrease of the Lyapunov coefficient revealed a true and important emergent effect which could not have been expected from the raw population abundance pattern.

Conclusions

Implications

The decreasing Lyapunov coefficients with increasing DCA concentration levels resembled a concentration-response relationship. This is an important finding as it is directly connected to the important species trait of reproductive capacity that strongly triggers the daphnids' population resilience (see discussion below). Unfortunately it is not straightforward to quantify the effect as the Lyapunov coefficient and the treatment level exhibit a reciprocal relationship. Furthermore, the upper effect bound is difficult to define, because Lyapunov coefficients approaching zero or values below zero mean a discrete change in system dynamics (phase transition towards a limit cycle or a stable equilibrium point respectively), not a steady change as required for a continuous concentration-response relationship. Thus the concentration-response relationship would exhibit discontinuities, difficult to interpret. In addition, Poincaré sections suggested that between 20 μg/l and 40 μg/l the attractor became unstable and a distinct transition to another attractor (basin of attraction) occurred. This would mean an extra discontinuity within the system's reaction to the increased treatment, which is a further clear contradiction to the concept of concentration-response relationships.

Nevertheless, a no-effect threshold value can easily be inferred from the distribution of Lyapunov coefficients. A simple test on significant differences between the mean values of controls and treatment levels (see Tab. 1) can be conducted. In the present study this threshold turned out to be smaller than 2.5 μg/l for all three age classes as well as the overall population (see Tab. 2). Such a threshold could serve as a relevant indicator to assess non-linear effects in population time-series data.

The population dynamics can generally be decomposed into a deterministic (periodic plus chaotic) and a stochastic part, the latter not being considered in this study. The authors are well aware about the diverse discussion about the problem how to distinguish stochastic fluctuations from chaotic dynamics (e.g. [96], [97], [98], [99], [100], [101]). To reduce sensitivity on stochastic fluctuations we used the function `lyap k` from the `Tisean` package which is said to suppress statistical fluctuations by excluding reference points which have no more than `nfmin` (default = 10) neighbours closer than ε [83].

The control level was found to exhibit a larger amount of chaotic dynamics than the treatments. Nevertheless, this does not mean that the controls were not predictable and thus not suitable for assessment. The dynamics produced by the IDAMP-model appeared clearly bounded and represented the naturally occurring

Table 2. *p*-values from Wilcoxon signed-rank tests on statistical differences between mean maximum Lyapunov coefficients per treatment level for the overall population.

	2.5 μg/l	5 μg/l	10 μg/l	20 μg/l	40 μg/l
Control	4.853E-4 *	3.489E-6 *	1.137E-6 *	2.267E-9 *	† *
2.5 μg/l		0.2401	0.1183	6.919E-3 *	† *
5 μg/l			0.5842	0.09201	† *
10 μg/l				0.316	† *
20 μg/l					† *

†: $p < 2.2e-16$, significant differences with $p < 0.05$ are marked by an asterisk.
doi:10.1371/journal.pone.0096270.t002

fluctuations within an ecologically stable population correctly, esp. under undisturbed conditions [30].

Natural populations as well as theoretical populations from ecosystem models are known to exist in a mixture of deterministic (periodical, ordered) and chaotic (irregular) dynamics [102], [103]. In our study it was shown that the ratio between the two deterministic parts of population dynamics (periodic vs. chaotic) increased with increasing disturbance resulting in the hypothesis that the capability for resilience decreased with increasing disturbance. As can be seen from Figs. 4 and 6 the dynamics are clearly bounded and the population got more and more trapped within a very small phase space region. From these findings it can be supposed that the population's flexibility to react to further external disturbance was reduced.

As can be concluded from the Poincaré maps used in this study, the DCA treatment seemed to destabilize the attractor above 20 µg DCA/l. Consulting the definition of ecological resilience, it can be assumed that this effect was caused by altering the phase space stability landscape, in the way that the system settled within a new basin of attraction. The fact that the system attractor became unstable could mean more than the gradually changing ratio of chaotic and periodic dynamics with increasing disturbance (see autocorrelation function and recurrence plot). It means a discrete, qualitative change in system dynamics (phase transition), which can be attributed to a shift in demographic parameters (like survival or fecundity) and has already been reported before for insect populations [104].

But, generally it is assumed that a press disturbance causes sustained alterations within a system's dynamics. A system under press disturbance is generally not expected to return to an initial configuration [16]. At least for the highest treatment tested it was supposed that there was an increased chance, that the system would not be able to return to the dynamics typical for less disturbed conditions simply by virtue of its own strength when the disturbance was removed. Instead it would need a further 'disturbance' to force the system back to the attractor which was observed for up to 20 µg/l due to the altered stability landscape. Recovery in this context would mean a reconfiguration of the stability landscape to a state typical for less disturbed systems. The problem about this definition within the experimental context is that for treatment levels up to 20 µg/l no change of the stability landscape was visible at all, whereas for 40 µg/l a change was found indeed. As a reconfiguration step (switching off the press disturbance) was not included within the experimental design, it was only possible to observe the potential for recovery, not an actual recovery event within the populations. But, from the findings in this study it was concluded that the chance for a change in attractor orientation increased with increasing treatment level.

Outlook

The study revealed that recovery and resilience in age-class structured populations exposed to environmental stress are complex matters and have to be assessed rather deeply to come to relevant conclusions.

Chaos was observed besides periodicity in the model-based age-structured *Daphnia* population. This was reported before for different models, populations under laboratory conditions and even for multi-species communities under laboratory and semi field conditions (e.g., [38], [95], [104], [105], [106], [107]). Our study connects this fact to the idea of a dynamical definition of recovery. In this study the amount of chaotic dynamics turned out to be the largest in the undisturbed control treatments, pointing out the importance of the natural mechanism of population stabilization via fluctuation in terms of resilience, defined as the flexibility to changes in environmental conditions (dynamical plasticity). The unpredictable part of population dynamics in the control treatments showed that the specification of recovery in terms of the return to the undisturbed control state maybe a questionable definition, as the theory behind the recovery concept is usually a static, not a dynamical one. Using Lyapunov coefficients as indicators it was shown that a disturbance reduces the amount of chaoticity or non-linear dynamics within the population. In our opinion this effect gives strong evidence that anthropogenic stressors can reduce the resilience of age-structured population and foster linear dynamics and resulting periodicity, reflecting equilibrium conditions.

Reduced resilience has a negative influence on populations' ability to respond to further disturbances and makes them more susceptible to extinction due to climatic changes, for example. This is an increasingly important aspect in ecological research which has been reported for many systems and their population, some of them highly endangered (e.g., [108], [109]).

These results stand in contrast to some other studies which instead suggest the increase of chaos and according decrease of stability in populations exposed to environmental stress [110]. But, in this study the constant press disturbance in combination with the discrete reproduction cycle of the daphnids results in a situation comparable to a periodic forcing which has already been shown to result in phase transitions in the dynamics of time discrete non-linear ecosystem models, either from periodic behaviour to chaos [111] or from chaotic dynamics to periodicity, the latter resulting in a chaos control mechanism [112].

The study at hand supports the hypothesis that chaotic oscillations reduce the degree of synchrony among populations or subdivisions of populations (decorrelation effect), thereby stabilizing the system and reducing the probability of extinction (chaos-mediated survival), an interpretation already been published for meta-populations before [113]. Thus, we think that our findings add an important aspect to the open discussion about resilience and chaotic population dynamics in the presence of environmental disturbance, which is still an exciting area of research [114].

The quantitative change in system dynamics and the two constituting components can effectively be visualised by linear methods (autocorrelation analysis) and non-linear techniques (recurrence plot, Poincaré section, Lyapunov exponents). Lyapunov coefficient proved to be a useful indicator to detect disturbance thresholds quantifying non-linear effects of contaminants within the *Daphnia* population. Even a concentration-response like relationship was observed for Lyapunov coefficients in this study. The modelling of non-linear effects is thus regarded as important by the authors, as non-linearities in species populations cannot be predicted by linear techniques in the long run.

The authors propose their methodology as a way to test for statistically significant non-linear effects of general disturbance on dynamics in long-term population data sets, especially when assessing the risk that a disturbance (like introducing chemicals in ecosystems) persistently alters the dynamics. It allows assessing an important emergent ecological effect on the population level which cannot be retrieved by standard assessment methods, neither based on individual endpoints like filtration rates nor based on population endpoints like growth rates.

The aim of this study was not to propose alternative definitions for the terms stability or recovery, nor to discuss the appearance of non-linear dynamics in population models. To come to a time explicit statement about recovery, like in the approach of engineering resilience, a larger number of simulation studies have

to be conducted, so that a mathematical probability can be computed for the fact whether a population dynamic time series returns to the control dynamics after a given time.

Further studies will be done in the future to find a suitable statistical representation for a recovery statement, elucidate the questions about a lasting altered fitness landscape induced by disturbance, calculate mathematical probabilities for this and use finer increments in the disturbance concentration levels to detect a possible point of attractor instability more precisely.

Supporting Information

Figure S1 Daphnia abundance from all 100 simulation runs. Solid line: mean abundance, dashed line: 95% confidence interval. Top panel: neonates, mid panel: juveniles, bottom panel: adults.
(TIFF)

Figure S2 Autocorrelation function of simulation run 1. From top to bottom: control, 2 µg/l, 5 µg/l, 10 µg/l, 20 µg/l, 40 µg/l, resp. Top panel: neonates, mid panel: juveniles, bottom panel: adults.
(TIFF)

Figure S3 Embedded time series of simulation run 1. From top left to bottom right: control, 2 µg/l, 5 µg/l, 10 µg/l, 20 µg/l, 40 µg/l, resp. ($d = 1$, $m = 2$). Top panel: neonates, mid panel: juveniles, bottom panel: adults.
(TIFF)

Figure S4 Recurrence plot of simulation run 1. From top left to bottom right, row-wise: control, 2 µg/l, 5 µg/l, 10 µg/l, 20 µg/l, 40 µg/l, resp. ($d = 1$, $m = 1,2$, $\varepsilon = 1$). Top panel: neonates, mid panel: juveniles, bottom panel: adults).
(TIFF)

Figure S5 Poincaré sections of simulation run 1. From top left to bottom right, row-wise: control, 2 µg/l, 5 µg/l, 10 µg/l, 20 µg/l, 40 µg/l, resp. ($d = 1$, $m = 3$, $C = 1$). Top panel: neonates, mid panel: juveniles, bottom panel: adults.
(TIFF)

MATLAB source code S1 Non-linear time series analysis using the Tisean package.
(DOC)

Acknowledgments

We thank our colleagues for comments and discussions during early stages of this study. We also thank the anonymous reviewers for their helpful comments.

Author Contributions

Conceived and designed the experiments: RO KS TGP MR. Performed the experiments: RO KS TGP MR. Analyzed the data: RO KS TGP MR. Contributed reagents/materials/analysis tools: RO KS TGP MR. Wrote the paper: RO KS TGP MR.

References

1. Millennium Ecosystem Assessment (MEA) (2005) Ecosystems and Human Well-being: Synthesis. Washington, DC: Island Press. 137 p.
2. Ottermanns R, Hopp PW, dos Santos GP, Meyer S, Roβ-Nickoll M (2011) Causal relationship between leaf litter beetle communities and regeneration patterns of vegetation in the Atlantic rainforest of Southern Brazil (Mata Atlântica). Ecological Complexity 8: 299–309.
3. Newton I, Wyllie I (1992) Recovery of a sparrowhawk population in relation to declining pesticide contamination. Journal of Applied Ecology 29: 476–484.
4. Chazdon RL (2003) Tropical forest recovery: legacies of human impact and natural disturbances. Perspectives in Plant Ecology, Evolution and Systematics 6: 51–71.
5. Barnthouse LW (2004) Quantifying population recovery rates for ecological risk assessment. Environmental Toxicology and Chemistry 23: 500–508.
6. Liess M, Pieters BJ, Duquesne S (2006) Long-term signal of population disturbance after pulse exposure to an insecticide: Rapid recovery of abundance, persistent alteration of structure. Environmental toxicology and chemistry 25: 1326–1331.
7. Hopp PW, Ottermanns R, Meyer S, Roβ-Nickoll M (2010) Recovery of litter inhabiting beetle assemblages during forest regeneration in the Atlantic Forest of Southern Brazil. Insect Conservation and Diversity 3: 103–113.
8. Wallace JB (1990) Recovery of lotic macroinvertebrate communities from disturbance. Environmental Management 14: 605–620.
9. Grimm V, Wissel C (1997) Babel, or the ecological stability discussions-an inventory and analysis of terminology and a guide for avoiding confusion. Oecologia 109: 323–34.
10. Jørgensen SE, Svirezhev YM (2004) Towards a Thermodynamic Theory for Ecological Systems. Amsterdam: Elsevier. 366 p.
11. Gunderson LH (2000) Ecological resilience-in theory and application. Annual Review of Ecology, Evolution, and Systematics 31: 425–39.
12. Webb CT (2007) What is the role of ecology in understanding ecosystem resilience? BioScience 57: 470–471.
13. Pimm SL (1984) The complexity and stability of ecosystems. Nature 307: 321–326.
14. Holling CS (1973) Resilience and stability of ecological systems. Annual Review of Ecology, Evolution, and Systematics 4: 1–23.
15. Perry DA, Amaranthus MP (1997) Disturbance, recovery, and stability. In: Kohm KA, Franklin JF, editors. Creating a Forestry for the 21st Century - The Science of Ecosystem Managament. Washington, DC: Island Press. pp. 31–56.
16. Newman MC, Clements WH (2008) Ecotoxicology: A Comprehensive Treatment. Boca Raton: CRC Press. 852 p.
17. White PS, Pickett STA (1985) The Ecology of Natural Disturbance and Patch Dynamics. New York: Academic Press. 488 p.
18. Bender EA, Case TJ, Gilpin ME (1984) Pertubation experiments in community ecology: Theory and practice. Ecology 65: 1–13.
19. Ricker WE (1954) Stock and recruitment. Journal of the fisheries research board of Canada 11: 559–623.
20. Caswell H (1989) Matrix Population Models, Construction, Analysis and Interpretation. Sunderland, MA: Sinauer Associates. 328 p.
21. Kareiva P (1989) Renewing the dialogue between theory and experiments in population ecology. In: Roughgarden J, May RH, Levin SA, editors. Perspectives in Ecological Theory. Princeton: Princeton University Press. pp. 68–88.
22. Tang S, Chen L (2002) Density-dependent birth rate, birth pulses and their population dynamic consequences. Journal of Mathematical Biology 44: 185–199.
23. Metz JAJ, Diekmann O (1986) The Dynamics of Physiologically Structured Populations. Lecture Notes in Biomathematics 68. Berlin: Springer. 73 p.
24. Ebenman B, Persson L (1988) Size-structured Populations: Ecology and Evolution. Berlin: Springer. 300 p.
25. Gurtin ME, MacCamy RC (1974) Nonlinear age-dependent population dynamics. Archive for Rational Mechanics and Analysis 54: 281–295.
26. Cushing JM (1987) Equilibria and oscillations in age-structured population growth models. In: Shukla JB, Hallam TG, Capasso V, editors. Mathematical Modelling of Environmental and Ecological Systems. New York: Elsevier. pp. 153–175.
27. Webb GF (1985) Theory of Nonlinear Age Dependent Population Dynamics. New York: Marcel Dekker. 312 p.
28. Higgins K, Hastings A, Botsford LW (1997) Density dependence and age structure: nonlinear dynamics and population behaviour. American Naturalist 149: 247–269.
29. Preuss TG, Hammers-Wirtz M, Hommen U, Rubach MN, Ratte HT (2009) Development and validation of an individual based Daphnia magna population model: The influence of crowding on population dynamics. Ecological Modelling 220: 310–329.
30. Preuss TG, Hammers-Wirtz M, Ratte HT (2010) The potential of individual based population models to extrapolate effects measured at standardized test conditions to relevant environmental conditions - an example for 3,4-dichloroaniline on Daphnia magna. Journal of Environmental Monitoring 12: 2070–2079.
31. Tang S, Chen L (2003) Multiple attractors in stage-structured population models with birth pulses. Bulletin of Mathematical Biology 65: 479–495.
32. Levin SA, Goodyear CP (1980) Analysis of an age-structured fishery model. Journal of Mathematical Biology 9: 245–274.
33. Levin SA (1981) Age-structure and stability in multiple-age spawning populations. In: Vincent TL, Skowrinski JM, editors. Lecture Notes in Biomathematics 40. Berlin: Springer. pp. 21–45.
34. Hastings A (1983) Age-dependent predation is not a simple process, I, continuous time models. Theoretical Population Biology 23: 347–362.

35. Hastings A (1984) Delay in recruitment at different trophic levels: effects on stability. Journal of Mathematical Biology 21: 35–44.
36. Bergh O, Getz WM (1988) Stability of discrete age-structured and aggregated delay-difference population models. Journal of Mathematical Biology 26: 551–581.
37. Aiello WG, Freedman HI (1990) A time-delay model of single-species growth with stage structure. Mathematical biosciences 101: 139–153.
38. Cushing JM, Costantino RF, Dennis B, Desharnais RA, Henson SM (1998) Nonlinear population dynamics: models, experiments and data. Journal of Theoretical Biology 194: 1–9.
39. Cushing JM (1984) Existence and stability of equilibria in age-structured population dynamics. Journal of Mathematical Biology 20: 259–276.
40. Ebenman B (1987) Niche differences between age classes and intraspecific competition in age-structured populations. Journal of Theoretical Biology 124: 25–33.
41. Liu L, Cohen JE (1987) Equilibrium and local stability in a logistic matrix model for age-structured populations. Journal of Mathematical Biology 25: 73–88.
42. Hastings A (1992) Age dependent dispersal is not a simple process: Density dependence, stability, and chaos. Theoretical Population Biology 41: 388–400.
43. Nisbet RM, Onyiah LC (1994) Population dynamic consequences of competition within and between age classes. Journal of Mathematical Biology 32: 329–344.
44. Stenseth NC, Bjørnstad ON, Saitoh T (1996) A Gradient from Stable to Cyclic Populations of Clethrionomys rufocanus in Hokkaido, Japan. Proceedings of the Royal Society of London Series B 263: 1117–1126.
45. Wikan A, Mjølhus E (1996) Overcompensatory recruitment and generation delay in discrete age-structured population models. Journal of Mathematical Biology 35: 195–239.
46. Bjørnstad ON, Nisbet RM, Fromentin J-M (2004) Trends and cohort resonant effects in age-structured populations. Journal of Animal Ecology 73: 1157–1167.
47. May RM, Conway GR, Hassell MP, Southwood TRE (1974) Time delays, density-dependence and single species oscillations. Journal of Animal Ecology 47: 249–267.
48. Hassell MP (1975) Density dependence in simple species populations. Journal of Animal Ecology 44: 263–295.
49. May RM (1975) Biological populations obeying difference equations: Stable points, stable cycles, and chaos. Journal of Theoretical Biology 49: 511–524.
50. Guckenheimer J, Oster G, Ipaktchi A (1977) The dynamics of density-dependent population models. Journal of Mathematical Biology 4: 101–147.
51. Tschumy WO (1981) Competition between juveniles and adults in age-structured populations. Theoretical Population Biology 21: 255–268.
52. Bellows TS (1982) Analytical models for laboratory populations of Callosobruchus chinensis and C. maculatus (Coleoptera, Bruchidae). Journal of Animal Ecology 51: 263–287.
53. Ebenman B (1988) Competition between age-classes and population dynamics. Journal of Theoretical Biology 131: 389–400.
54. Cushing JM, Li J (1989) On Ebenman's model for the dynamics of a population with competing juveniles and adults. Bulletin of Mathematical Biology 51: 687–713.
55. Cushing JM, Li J (1992) Intra-specific competition and density-dependent juvenile growth. Bulletin of Mathematical Biology 54: 503–519.
56. Adema DMM (1978). Daphnia magna as a test animal in acute and chronic toxicity tests. Hydrobiologia 59: 125–134.
57. Dodson SI, Hanazato T (1995) Commentary on effects of anthropogenic and natural organic-chemicals on development, swimming behavior, and reproduction of Daphnia, a key member of aquatic ecosystems. Environmental Health Perspective 103: 7–11.
58. Lampert W (2006) Daphnia: model herbivore, predator and prey. Polish Journal of Ecology 54: 607–620.
59. Baudo R (1987) Ecotoxicological testing with Daphnia. In: Peters, R.H., de Bernardi, R., Daphnia. Mem. Ist. Ital. Idrobiol. 45, 461–482.
60. Peters R, De Bernardi R (1987) Daphnia. Pallanza: Verbania. 399 p.
61. Wogram J, Liess M (2001) Rank ordering of macroinvertebrate species sensitivity to toxic compounds by comparison with that of Daphnia magna. Bulletin of Environmental Contamination and Toxicology 67: 360–367.
62. Berge WF ten (1978) Breeding Daphnia magna. Hydrobiologia 59: 121–123.
63. European Commission (EU) (2006) 3,4-DICHLOROANILINE (3,4-DCA), Summary Risk Assessment Report, Institute for Health and Consumer Protection. Ispra: European Chemicals Bureau (IHCP). 144 p.
64. Elendt B-P (1990) Influence of water composition on the chronic toxicity of 3,4-dichloroaniline to Daphnia magna. Water Research 24: 1169–1172.
65. Baird DJ, Barber I, Bradley M, Soares AMVM, Calow P (1991) A comparative study of genotype sensitivity to acute toxic stress using clones of Daphnia magna straus. Ecotoxicology and Environmental Safety 21: 257–265.
66. Klüttgen B, Kuntz N, Ratte HT (1996) Combined effects of 3,4-dichloroaniline and food concentration on life-table data of two related cladocerans, Daphnia magna and Ceriodaphnia quadrangular. Chemosphere 32: 2015–2035.
67. Adema DMM, Vink GJ (1981) A comparative study of the toxicity of 1,1,2-Trichloroethane, Dieldrin, Pentachlorphenol and 3,4-Dichloroaniline for marine and fresh water organisms. Chemosphere 10: 533–544.
68. van der Hoeven N (1990) Effect of 3,4-dichloroaniline and metavanadate on Daphnia populations. Ecotoxicology and Environmental Safety 20: 53–70.
69. Box GEP, Jenkins GM (1976) Time series analysis forecasting and control. San Francisco: Holden-Day. 575 p.
70. Takens F (1981) Detecting strange attractors in turbulence. In: Rand DA, Young L-S. Dynamical Systems and Turbulence. Lecture Notes in Mathematics 898. Berlin: Springer. pp. 366–381.
71. Mañé R (1981) On the dimension of the compact invariant sets of certain nonlinear maps. In: Rand DA, Young L-S. Dynamical Systems and Turbulence. Lecture Notes in Mathematics 898. Berlin: Springer. pp. 230–242.
72. Eckmann J-P, Ruelle D (1985) Ergodic theory of chaos and strange attractors. Reviews of Modern Physics 57: 617–656.
73. Sauer T, Yorke JA, Casdagli M (1991) Embedology. Journal of Statistical Physics 65: 579–616.
74. Abarbanel HDI, Carro lTA., Pecora LM, Sidorowich JJ, Tsimring S (1994) Predicting physical variables in time-delay embedding. Physical Review E 49: 1840–1853.
75. Eckmann J-P, Kamphorts SO, Ruelle D (1987) Recurrence Plots of Dynamical Systems. Europhysics Letters 4: 973–977.
76. Ott E (2002) Chaos in Dynamical Systems. Cambridge: Cambridge University Press. 492 p.
77. Argyris JH, Faust G, Haase M (1994) An Exploration of Chaos. Amsterdam: North-Holland. 772 p.
78. Marwan N, Wessel N, Meyerfeldt U, Schirdewan A, Kurths J (2002) Recurrence-plot-based measures of complexity and their application to heart-rate-variability data. Physical Review E 66: 026702.
79. Poincaré H (1892) Les Méthods Nouvelles de la Mécanique Céleste. Paris: Gauthier-Villars. 429 p.
80. Kantz H (1994) A robust method to estimate the maximal Lyapunov exponent of a time series. Physics Letters A 185: 77–87.
81. Rosenstein MT, Collins JJ, De Luca CJ (1993) A practical method for calculating largest Lyapunov exponents from small data sets. Physica D 65: 117–134.
82. Wolf A, Swift JB, Swinney HL, Vastano JA (1985) Determining Lyapunov exponents from a time series. Physica D 16: 285–317.
83. Kantz H., Schreiber T (2004) Nonlinear time series analysis, 2nd edition, Cambridge: Cambridge University Press. 367 p.
84. Huston M, DeAngelis D, Post W (1988) New computer models unify ecological theory. BioScience 38: 682–691.
85. Naito W, Miyamoto K-I, Nakanishi J, Masunaga S, Bartell SM (2002) Application of an ecosystem model for aquatic ecological risk assessment of chemicals for a Japanese lake. Water Research 36: 1–14.
86. Lopes C, Pery ARR, Chaumot A, Charles S (2005) Ecotoxicology and population dynamics: Using DEBtox models in a Leslie modeling approach. Ecological Modelling 188: 30–40.
87. van den Brink PJ, Brown CD, Dubus IG (2006) Using the expert model PERPEST to translate measured and predicted pesticide exposure data into ecological risks. Ecological Modelling 191: 106–117.
88. van den Brink PJ, Baveco JM, Verboom J, Heimbach F (2007) An individual-based approach to model spatial population dynamics of invertebrates in aquatic ecosystems after pesticide contamination. Environmental Toxicology and Chemistry 26: 2226–2236.
89. Gergs A, Zenker A, Grimm V, Preuss TG (2013) Chemical and natural stressors combined: from cryptic effects to population extinction. Scientific Reports 3: 2036.
90. Gabsi F, Hammers-Wirtz M, Grimm V, Schäffer A, Preuss TG (2014): Coupling different mechanistic effect models for capturing individual- and population-level effects of chemicals: Lessons from a case where standard risk assessment failed. Ecological Modelling 280: 18–29.
91. Hegger R, Kantz H, Schreiber T (1999) Practical implementation of non linear time series methods: The TISEAN Package. Chaos 9: 413–435.
92. R Core Team (2013) R: A Language and Environment for Statistical Computing. Vienna: R Foundation for Statistical Computing. Available: http://www.R-project.org. Accessed 18 September 2013.
93. Gao J, Hu J, Tung W-W, Cao Y, Sarshar N, et al. (2006) Assessment of long-range correlation in time series: How to avoid pitfalls. Physical Review E 73: 016117.
94. Marwan N, Romano MC, Thiel M, Kurths J (2007) Recurrence plots for the analysis of complex systems. Physics Reports 438: 237–329.
95. Costantino RF, Desharnais RA, Cushing JM, Dennis B (1997) Chaotic Dynamics in an Insect Population. Science 275: 389–391.
96. Zunino L, Soriano MC, Rosso OA (2012) Distinguishing chaotic and stochastic dynamics from time series by using a multiscale symbolic approach. Physical Review E 86: 04621.
97. Gao JB, Hu J, Tung WW, Cao YH (2006) Distinguishing chaos from noise by scale-dependent Lyapunov exponent. Physical Review E 74: 066204 s2006d.
98. Dennis B, Desharnais RA, Cushing JM, Henson SM, Costantino RF (2003) Can noise induce chaos? Oikos 102: 329–339.
99. Ellner S, Turchin T (1995) Chaos in a noisy world: new methods and evidence from time-series analysis. American Naturalist 145: 343–375.
100. Nychka D, Ellner S, Gallant AR, McCaffrey D (1992) Finding chaos in noisy systems. Journal of the Royal Statistical Society B 54: 399–426.
101. Kostelich EJ, Yorke JA (1990) Noise reduction: Finding the simplest dynamical system consistent with the data. Physica D: Nonlinear Phenomena 41: 183–196.

102. Turchin P, Ellner SP (2000) Living on the edge of chaos: population dynamics of Fennoscandian voles. Ecology 81: 3099–3116.
103. Golestani A, Gras R (2010) Regularity analysis of an individual-based ecosystem simulation. Chaos 20: 043120.
104. Costantino RF, Cushing JM, Dennis B, Desharnias RA (1995) Experimentally induced transitions in the dynamic behaviour of insect populations. Nature 375: 227–230.
105. Dennis B, Desharnais RA, Cushing JM, Henson SM, Costantino RF (2001) Estimating chaos and complex dynamics in an insect population. Ecological Monographs 71: 277–303.
106. Becks L, Hilker FM, Malchow H, Jürgens K, Arndt H (2005) Experimental demonstration of chaos in a microbial food web. Nature 435: 1226–1229.
107. Benincà E, Huisman J, Heerkloss R, Jöhnk KD, Branco P, et al. (2008) Chaos in a long-term experiment with a plankton community. Nature 451: 822–825.
108. Hughes TP, Baird AH, Bellwood DR, Card M, Connolly SR, et al. (2003) Climate change, human impacts, and the resilience of coral reefs. Science 301: 929–933.
109. Ling SD, Johnson CR, Frusher SD, Ridgway KR (2009) Overfishing reduces resilience of kelp beds to climate-driven catastrophic phase shift. PNAS 106: 22341–2345.
110. Allen JC (1990) Factors contributing to chaos in population feedback systems. Ecological Modelling 51: 281–298.
111. Summers D, Cranford JG, Healey BP (2000) Chaos in periodically forced discrete-time ecosystem models. Chaos, Solutions and Fractals 11: 2331–2342.
112. Vandermeer J, Stone L, Blasius B (2001) Categories of chaos and fractal basin boundaries in forced predator-prey models. Chaos, Solutions and Fractals 12: 265–276.
113. Allen JC, Schaffer WM, Rosko D (1993) Chaos reduces species extinction by amplifying local population noise. Nature 364: 229–232.
114. Newman MC (2013) Quantitative Ecotoxicology, 2nd edition. Boca Raton: CRC Press. 570 p.
